# Multidisciplinary management of disseminated *Exophiala dermatitidis* mycosis in an infant with mixed phenotype acute leukemia: a case report

**DOI:** 10.1186/s12879-022-07773-w

**Published:** 2022-10-23

**Authors:** Ryo Nakatani, Miho Ashiarai, Hiroki Yoshihara, Keigo Yada, Taiki Nozaki, Takeshi Ushigusa, Nobuyoshi Mori, Daisuke Hasegawa

**Affiliations:** 1 Department of Pediatrics, St. Luke’s International Hospital, 9-1, Akashi-Cho, Chuo-Ku, Tokyo, Japan; 2 Department of Pediatric Surgery, St. Luke’s International Hospital, Tokyo, Japan; 3 Department of Radiology, St. Luke’s International Hospital, Tokyo, Japan; 4 Department of Pathology, St. Luke’s International Hospital, Tokyo, Japan; 5 Department of Infectious Diseases, St. Luke’s International Hospital, Tokyo, Japan

**Keywords:** *Exophiala dermatitidis*, Infant, Acute leukemia, Ecthyma gangrenosum, Ethanol lock therapy

## Abstract

**Background:**

*Exophiala dermatitidis* is a dematiaceous fungus isolated from various environmental sources. Systemic *E. dermatitidis* infections can lead to fatal outcomes, and treatment has not yet been standardized. Although *E. dermatitidis* is also known to cause cutaneous infection, it has not been previously reported to appear as ecthyma gangrenosum (EG), an uncommon cutaneous lesion in neutropenic patients that is mainly caused by *Pseudomonas aeruginosa*.

**Case presentation:**

A 2-month-old male infant with mixed-phenotype acute leukemia presented with prolonged fever unresponsive to antibacterial and antifungal agents during myelosuppression due to remission induction therapy. He also presented with skin lesions on the left wrist and left lower quadrant of the abdomen. The abdominal lesion gradually turned black and necrotic, which was consistent with the findings of the EG. *E. dermatitidis* was isolated from the blood, stool, wrist skin, and endotracheal aspirate. During hematopoietic recovery, consolidation in both lungs was evident. Multiagent antifungal treatment failed to eliminate *E. dermatitidis* from blood. In order to salvage the central venous catheter, ethanol lock therapy (ELT) was adopted, following which the blood culture became negative. The abdominal lesion that evolved as a necrotic mass connecting the small intestine and subcutaneous tissue adjacent to the skin was surgically resected. After these interventions, the general condition improved.

**Conclusion:**

Disseminated *E. dermatitidis* mycosis in the neutropenic infant was successfully managed with a multidisciplinary treatment consisting of multiagent antifungal treatment, ELT, and surgery.

## Background

*Exophiala* species are dematiaceous fungi characterized by their dimorphic character, switching from a yeast-like to a hyphal state. These species are distributed worldwide and has been isolated from humid environments such as soil, dishwashers, saunas, and bathrooms [1]. Among *Exophiala* species, *E. dermatitidis* is the most common human pathogen causing phaeohyphomycosis, an infection caused by several dark, melanin-pigmented dematiaceous fungal species. Phaeohyphomycosis is rare but remains important because of the ability to cause serious diseases such as dermatitis, pneumonia, cerebral abscess, and fungemia [2, 3].

Ecthyma gangrenosum (EG) is an uncommon cutaneous infection that occurs in critically ill, immunocompromised, or neutropenic patients. EG initially manifests as macular erythematous lesions that later become hemorrhagic and transform into tender erythematous areas with necrotic centers. *Pseudomonas aeruginosa* has been reported to be responsible for 70–90% of EG [4, 5], and the remaining cases are due to other microorganisms such as *Aeromonas hydrophila* [6] and *Fusarium oxysporum* [7]. Although *E. dermatitidis* is also known to cause cutaneous infections, it has not been reported as a causative agent of EG.

Herein, we report a case of mixed-phenotype acute leukemia (MPAL) who developed phaeohyphomycosis accompanied with fungemia, EG, and pneumonia due to *E. dermatitidis* during profound myelosuppression.

## Case presentation

A previously healthy 2-month-old male infant showed poor feeding and had fever. The abdomen was distended, the liver was palpated 5 cm below the right costal margin, and the spleen was palpated 5 cm below the left costal margin. Laboratory findings were as follows: white blood cell count 439 × 10^9^/L (neutrophils 0%, lymphocytes 2.0%, monocytes 1.0%, blasts 97.0%), hemoglobin 4.9 g/dL, platelet count 39 × 10^9^/L, creatinine 0.84 mg/dL, uric acid 11.5 mg/dL, and C-reactive protein (CRP) 0.07 mg/dL. Abdominal enhanced computed tomography (CT) revealed massive hepatosplenomegaly, bilateral kidney enlargement, and bowel wall thickening. The blast cells displayed two distinct cytomorphological populations: lymphoblasts and monoblasts. Immunophenotypic analysis showed that the blast cells commonly expressed CD19, CD15, and CD65. One third of the cells showed additional expression of CD20 and CD79a, while approximately 40% of the cells co-expressed CD14 and CD11b. Reverse transcription-polymerase chain reaction revealed *KMT2A-MLLT10* fusion transcript. The patient was diagnosed with MPAL and remission induction therapy was initiated according to the Interfant-06 protocol [8].

**Fig. 1:**
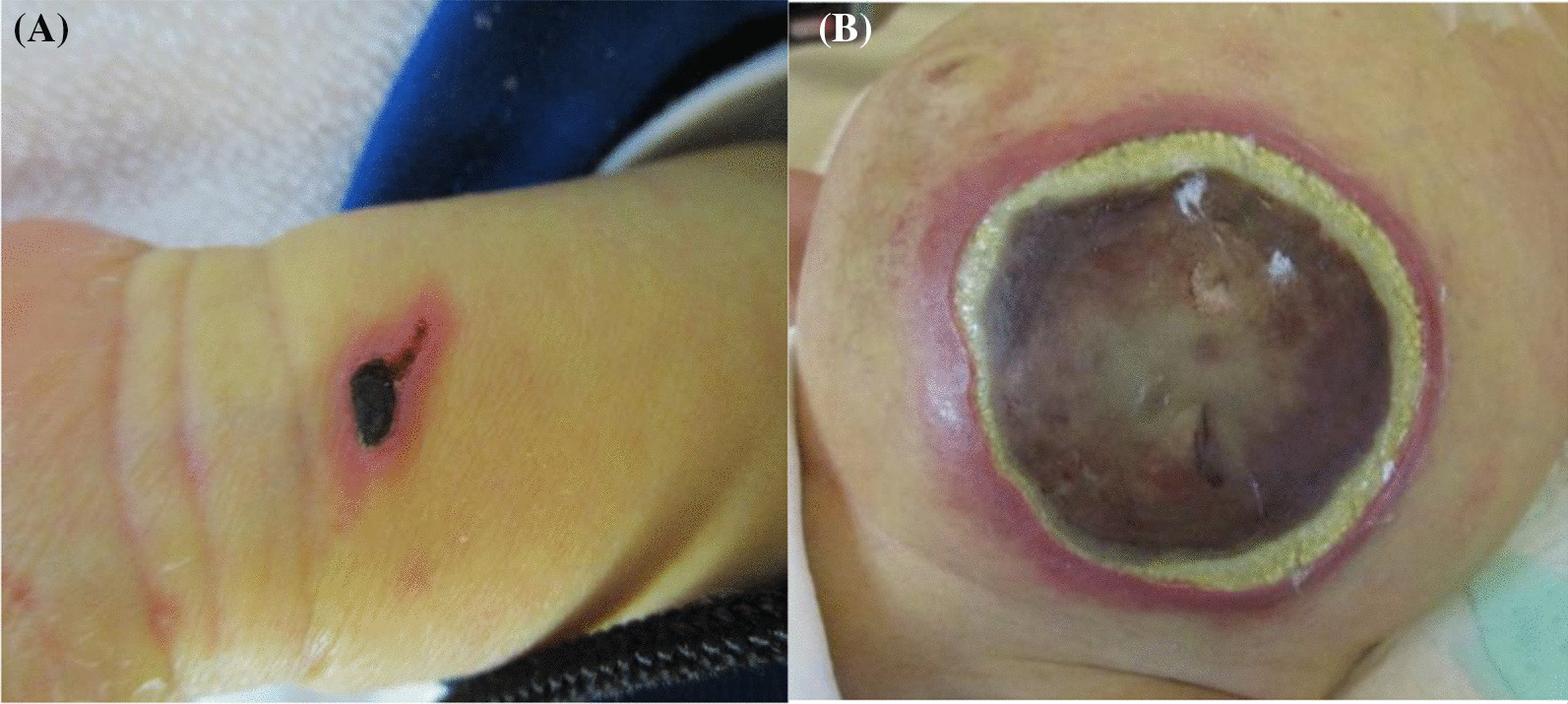
**A** Ecthyma gangrenosum on the left wrist, where the atrial line was inserted. **B** The edges of the ecthyma gangrenosum of the left lower quadrant of the abdomen that turned yellow after recovery of neutrophil counts

**Fig. 2:**
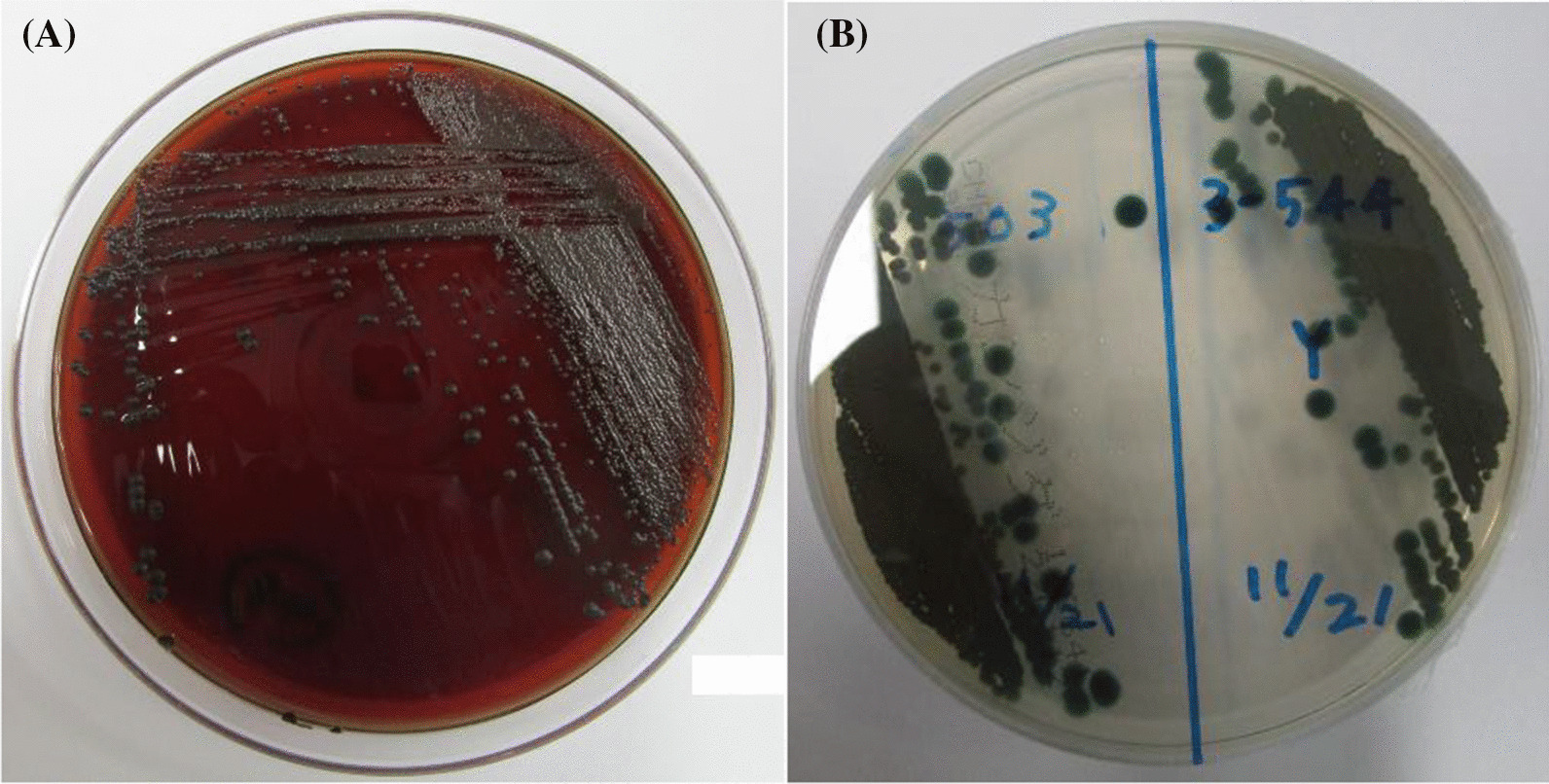
Colony of *Exophiala dermatitidis* on blood agar **A** and potato dextrose agar (**B**)

**Fig. 3:**
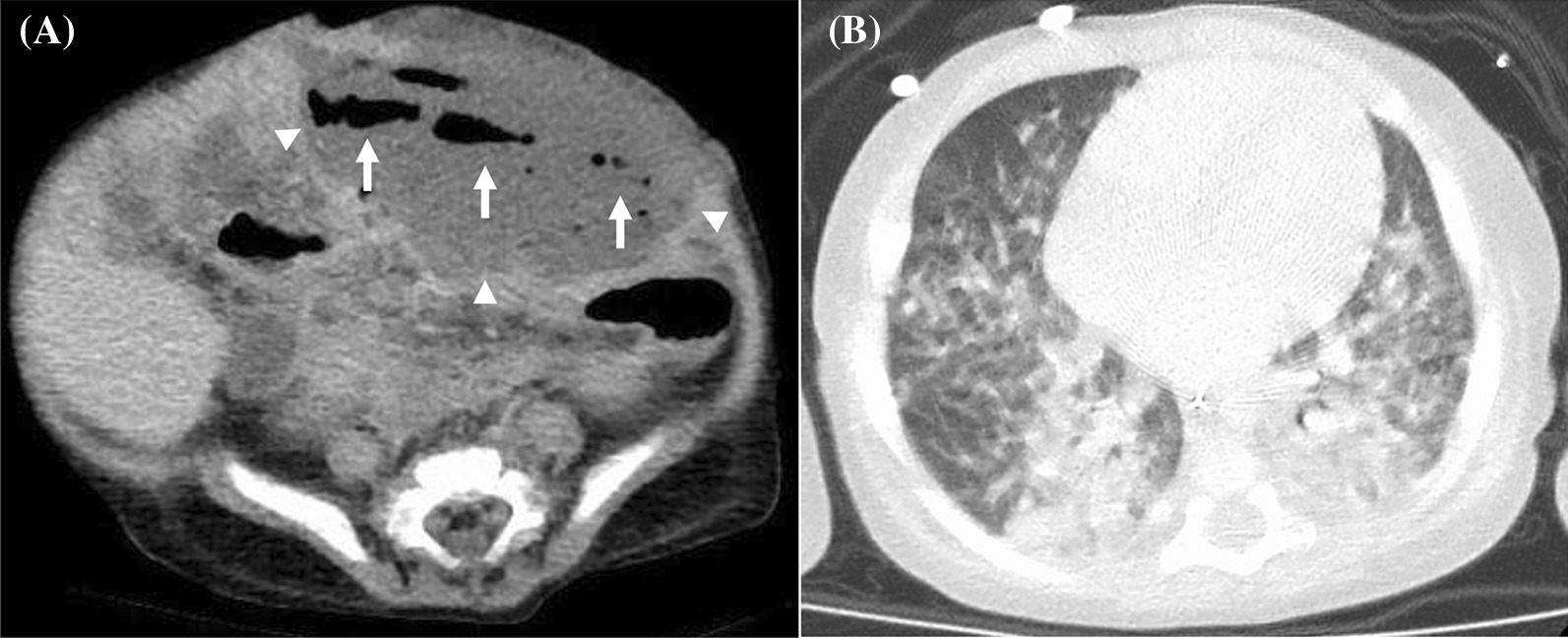
**A** Contrast-enhanced abdominal computed tomography showing extensive irregular necrotic mass (arrowheads) with gas (arrows), connecting with the small intestine and subcutaneous tissue. **B** Chest CT showing consolidations and multiple nodules in both lungs

**Fig. 4:**
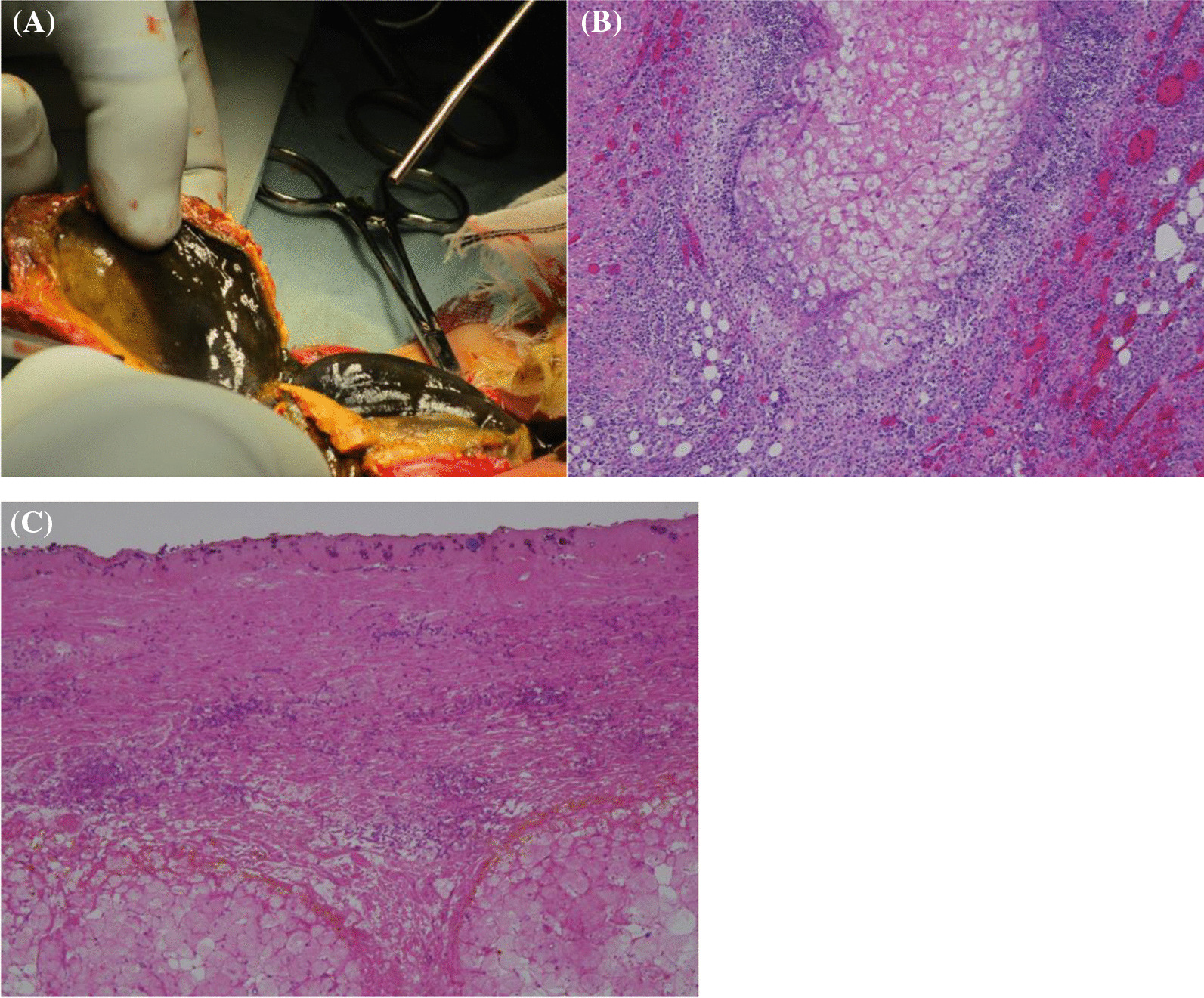
**A** Intraoperative findings showing fusion of the skin and intestinal tract, and part of the small bowel being necrotic. **B** Photomicrograph depicting granulomas around the necrotic perforation in the ileum mucosa. **C** Photomicrograph showing tissue necrosis in all layers of the abdominal wall

Although massive hepatosplenomegaly progressively worsened and renal and intestinal blood flow decreased, these findings were mitigated after the initiation of therapy. Despite intensive care and administration of rasburicase, the patient developed tumor lysis syndrome. Renal function was maintained without renal replacement therapy, but he developed severe acute encephalopathy and late seizures that required phenobarbital (PB) and levetiracetam. Despite blast cells infiltrating the cerebrospinal fluid (CSF), bacteria and fungi were not detected in the CSF. The blast cells responded well to treatment and became undetectable in the peripheral blood on day 14. The fever decreased in accordance with the blast reduction; however, the fever again increased on day 11. With the administration of meropenem, vancomycin, and micafungin [MCFG; 6 mg/kg intravenously (IV) every 24 h], the fever gradually decreased. On day 14, CRP and beta-D-glucan levels increased up to 23.65 mg/dL and 105.6 pg/mL, respectively, and the fever again increased on day 18. As yeast was detected in the blood culture on day 25, MCFG was switched to liposomal amphotericin B (L-AMB; 5 mg/kg IV, every 24 h) on day 27. Based on the echocardiographic findings, endocarditis was ruled out. Skin redness was observed in the left lower quadrant (LLQ) of the abdomen and left wrist, where the arterial line had previously been placed. The wrist lesion was covered with crust (Fig. 1A). The cultured blood specimen showed smooth and waxy yeast-like colonies appearing on potato dextrose agar and turned black on day 32 (Fig. 2), suggesting the presence of *Exophiala* species. Sequence analysis of the internal transcribed spacer region confirmed *E. dermatitidis* and the fungus was also detected in the skin of the left wrist, stool, and endotracheal aspirate. However, *E. dermatitidis* was not detected from the skin culture from the abdominal lesion. The patient was diagnosed with fungemia and skin infection due to *E. dermatitidis,* and voriconazole (VRCZ) was added at a dose of 9 mg/kg IV and administered every 12 h; the dosage was further adjusted based on the results of therapeutic drug monitoring. Despite L-AMB and VRCZ administration and recovery of normal hematopoiesis, *E. dermatitidis* was repeatedly detected in the blood cultures. However, we were hesitant to remove the tunneled central venous catheter (CVC) due to multiple drug administrations for systemic management and difficulty in gaining vascular access. After approval by the Institutional Ethics Board, we initiated ethanol lock therapy (ELT) as a catheter salvage strategy on day 43. Both lumens of the tunneled catheter were instilled with a dose of 1 mL of 25% ethanol for 1–2 h every day [9]. Blood culture became negative 4 days after the commencement of ELT. Although the skin lesion on the left wrist improved without debridement, the abdominal lesion gradually enlarged and became black, reaching a diameter of approximately 8 cm. The edge of the lesion turned yellow, and the center was necrotized, which was consistent with the findings of the EG (Fig. 1B). Contrast-enhanced thoraco-abdominal CT showed an extensive necrotic mass with gas in the left lower abdomen connected to the small intestine and subcutaneous tissue adjacent to the skin (Fig. 3A). Although consolidations and multiple nodules were also observed in both lungs (Fig. 3B), the respiratory condition was stable without the need for mechanical ventilation. The EG of the LLQ of the abdomen and adjacent necrotic intestine was resected *en bloc*, without complications, on day 52 (Fig. 4A). Intraoperatively, beneath the transmural necrosis of the abdominal wall, there were two perforations in the necrotic small intestine that required resection and oral jejunostomy/distal ileostomy. Because the adjacent sigmoid colon was also segmentally necrotized, the necrotic lesion was resected and end-to-end anastomosis was performed. Massive defects of the skin and abdominal wall were managed using negative-pressure wound therapy (Renasys-GO™, Smith & Nephew GmbH). Histological examination showed that the resected small intestine almost lacked a mucosal layer structure, and Grocott-methenamine-silver-staining-positive fungi were found (Fig. 4B). Subcutaneous adipose tissue showed fat necrosis, and numerous fungi were also observed in the skin lesions. (Fig. 4C). On day 53, caspofungin (CPFG; 50 mg/m^2^ IV, every 24 h) was added because the isolated strain showed susceptibility to CPFG (the minimum inhibitory concentration being 0.25 μg/mL), and the trough level of VRCZ remained undetectable despite increasing the administration dose even after discontinuation of PB on day 61. The patient became afebrile on day 61 and his general condition improved. The highest value of beta-D-glucan (17,980 pg/mL) was seen on day 63, and the levels decreased thereafter. On day 66, the stomata and muscle layers were closed without complications. Subsequently, the skin defect was closed on day 107. ELT was continued until the removal of the CVC on day 72, without recurrence of fungemia. No fungi were detected at the tip of the CVC. The CRP level became normal just before removing the CVC, following which the administration of VRCZ, L-AMB, and CPFG was continued.

## Discussion and conclusions

The cause of disseminated *E. dermatitidis* mycosis involving fungemia, EG, and pneumonia in the current MPAL case may be multifactorial. Remarkable hepatosplenomegaly may have caused intestinal ischemia, making the tissue vulnerable to infections. Interfant-06 therapy targeting both myeloid and lymphoid cells is indispensable for MPAL. In this case though the patient responded well, it resulted in severe neutropenia and lymphocytopenia. The patient developed fungemia with skin lesions during prolonged and profound neutropenia and lymphocytopenia. We initially suspected candidiasis, but the color of the colony and biphasic development suggested *Exophiala* species; the molecular analysis then confirmed *E. dermatitidis*. Considering that *E. dermatitidis* was also detected in the skin of the left wrist, stool, and endotracheal aspirate, we speculate that *E. dermatitidis* invaded through small erosion of the previous arterial line in the left wrist, progressed systemically as fungemia, developed pneumonia, colonized the intestine with ischemic damage, and then extended to the surrounding skin. Another possible explanation may be that *E. dermatitidis* in the environment caused catheter-related bloodstream infection and then invaded the skin and adjacent intestines.

The previous study had reported that 8 out of 25 adult patients with systemic *E. dermatitidis* infection had a fatal outcome [10]. The outcome in immunocompromised hosts differs slightly; it was reported that 4 out of 5 adult patients with hematological malignancies survived after disseminated *E. dermatitidis* infection [11]. In comparison, *E. dermatitidis* infections in children are rare. Thirteen pediatric cases aged < 15 years have been previously reported (Table 1) [12–24]. The median age at diagnosis was 3.5 years (range 0.2–11). The underlying diseases included acute leukemia (n = 3), primary immunodeficiency (n = 1), human immunodeficiency virus infection (n = 1), and cystic fibrosis (n = 1). However, 5 children had no underlying conditions for fungal infection. Seven out of 8 children with underlying diseases developed fungemia and pneumonia, and 7 survived with antifungal agents with or without catheter removal [12–15, 22, 24]. The present case of MPAL also developed fungemia, pneumonia, and severe skin infection. Although multiagent antifungal treatment failed to improve the condition, the patient was successfully treated with ELT and surgical intervention. Notably, all the children without underlying conditions did not survive. It is difficult to draw conclusions from the limited number of case reports, and further studies are required to clarify the prognostic factors for *E. dermatitidis* infection.

**Table 1:** Summary of the 14 pediatric cases of *Exophiala dermatitidis* infection reported in literature

No	Age	Gender	Clinical manifestation	Undelying disease	Susceptibility MIC (µg/mL)	Treatment	Outcome	References
1	6	F	Pneumonia	Cystic fibrosis	No data	ITCZ	Survive	[12]
2	5	M	Nosocomial intravascular infection	ALL	No data	ITCZ	Survive	[13]
3	3	M	Fungemia	ALL	No data	Catheter removal, AMB, 5-FC	Survive	[14]
4	3	M	Catheter-associated fungemia	HIV infection	No data	Catheter removal, AMB, ITCZ	Survive	[15]
5	3	M	Intracranial hypertension, paraplegia	None	No data	FLCZ, ITCZ, L-AMB	Death	[16]
6	11	F	Liver cirrhosis	None	AMB 1; ITCZ 0.064; VRCZ 0.016	VRCZ	Death	[17]
7	8	M	Systemic lymphadenitis	None	No data	AMB, VRCZ	Death	[18]
8	3	M	Cerebritis	None	No data	AMB, ITCZ, 5-FC	Death	[19]
9	8	M	Brain abscess	None	No data	AMB, VRCZ, 5-FC	Death	[20]
10	8	M	Pneumonia	AML	No data	PSCZ	Death	[21]
11	2	F	Catheter-related bloodstream infection	Retroperitoneal teratoma	AMB 0.5; FLCZ 4; ITCZ 0.25; MCFG 2; VRCZ 0.12; 5-FC > 16	Catheter removal, L-AMB	Survive	[22]
12	4	F	Lymphadenitis, hemiparesis	Inherited *CARD9* deficiency	No data	L-AMB, VRCZ	Survive	[23]
13	0.2	F	Catheter-associated fungemia	Mitochondriopathy	AMB 1; CPFG 2; FLCZ 4; ITCZ ≤ 0.03; MCFG 2; PSCZ 0.06; VRCZ ≤ 0.03; 5-FC 4	Catheter removal, FLCZ	Survive	[24]
14	0.2	M	Ecthyma gangrenosum, fungemia, pneumonia	MPAL	AMB 2; CPFG 0.25; FLCZ 8; ITCZ 0.25; MCFG 1; VRCZ 0.12; 5-FC 4	CPFG, L-AMB, VRCZ, ELT, Surgery	Survive	Present case

*ALL* acute lymphoblastic leukemia, *AML* acute myeloid leukemia, *AMB* amphotericin B, *CPFG* caspofungin, *ELT* ethanol lock therapy, *FLCZ* fluconazole, *HIV* human immunodeficiency virus, *ITCZ* itraconazole, *L-AMB* liposomal amphotericin B, *MCFG* micafungin, *MPAL* mixed-phenotype acute leukemia; *PSCZ* posaconazole, *VRCZ* voriconazole, *5-FC* 5-fluorocytosine

To date, the treatment of *E. dermatitidis* has not been standardized. Based on previous reports, we administered L-AMB and VRCZ [25, 26]. Although posaconazole is also a therapeutic choice for phaeohyphomycosis, it is recommended only for children 2 years and above. A combinational antifungal therapy failed to eliminate *E. dermatitidis* from blood culture for several reasons. First, the trough level of VRCZ remained suboptimal even after the discontinuation of PB, which was assumed to result from rapid metabolism. In addition, *Exophiala* species. are known to form biofilms. Catheter removal may be recommended in cases with protracted fungemia; however, a catheter salvage strategy was considered in our case because of multiple drug administrations for systemic management and difficulty in vascular access. ELT has broad antimicrobial activity, including antifungal [27], and has been shown to affect biofilms in vitro; therefore, it is considered as an alternative treatment to conventional antibiotic therapy for catheter-related bloodstream infections. ELT has also been reported to be effective in children, including in hemato-oncology patients [27, 28]. Given that the blood culture became negative 4 days after the initiation of ELT in our case, ELT can be considered a promising option for managing refractory fungemia with CVC.

EG has been reported in both adults and children and occurs mostly in immunocompromised individuals. A review of 167 previously reported cases of EG showed that *P. aeruginosa* was detected in 74% of patients, and the remaining were caused by other microorganisms, including *Pseudomonas maltophilia* and *Aeromonas hydrophila* [4]. Fungal infections, such as *Mucor pusillus* and *Candida albicans* have also been reported to cause EG; however, so far, *Exophiala* species have not been reported as the causative agent. An 8-year-old male with acute myeloid leukemia undergoing allogeneic stem cell transplantation developed severe *E. dermatitidis* infection and bullous epidermolysis in which skin lesion became black, but the diagnosis of EG was not confirmed [21]. The Infection Working Group of the Italian Pediatric Hematology-Oncology Association reviewed 38 children with malignancies or bone marrow failure syndromes who developed EG [29]. *P. aeruginosa* was the major cause of EG in the pediatric hematology-oncology cohort, whereas no fungi were isolated. The perineal region is the most involved site; however, various parts of the body, including the face and trunk can also be affected [4, 29]. Although the mainstay of management for EG is antibiotic therapy, 60–70% of EG cases require surgical intervention [4, 29]. Because the EG in the abdomen in our case was large and the adjacent intestines were also involved, *en bloc* resection of the EG and intestines was required. A large defect in the abdominal wall was successfully managed with two-step closure and negative pressure wound therapy. We believed that this aggressive resection may have contributed to the source control.

In conclusion, *E. dermatitidis* caused severe systemic infections, including fungemia, pneumonia, and EG, in an infant with MPAL receiving intensive chemotherapy. Although disseminated *E. dermatitidis* mycosis can be fatal, we successfully managed it with multidisciplinary treatment consisting of multiagent antifungal treatment, ELT, and surgery. Nevertheless, to establish the optimal treatment for disseminated *Exophiala* mycosis, further studies are required.

## Data Availability

Not applicable.

## Abbreviations

CPFG: Caspofungin
CRP: C-reactive protein
CSF: Cerebrospinal fluid
CT: Computed tomography
CVC: Central venous catheter
EG: Ecthyma gangrenosum
ELT: Ethanol lock therapy
IV: Intravenously
L-AMB: Liposomal amphotericin B
LLQ: Left lower quadrant
MPAL: Mixed-phenotype acute leukemia
PB: Phenobarbital
VRCZ: Voriconazole
